# Combined effect of non-bacteriolytic antibiotic and inhibition of matrix metalloproteinases prevents brain injury and preserves learning, memory and hearing function in experimental paediatric pneumococcal meningitis

**DOI:** 10.1186/s12974-018-1272-8

**Published:** 2018-08-21

**Authors:** Lukas Muri, Denis Grandgirard, Michelle Buri, Michael Perny, Stephen L. Leib

**Affiliations:** 1 Neuroinfection Laboratory, Institute for Infectious Diseases, University of Bern, Friedbühlstrasse 51, 3001 Bern, Switzerland; 2 Graduate School for Cellular and Biomedical Sciences (GCB), University of Bern, Freiestrasse 1, 3012 Bern, Switzerland

**Keywords:** Pneumococcal meningitis, Brain injury, Neurologic sequelae, Neuroinflammation, Hearing loss, Combined adjuvant therapy

## Abstract

**Background:**

Pneumococcal meningitis is associated with high mortality and morbidity rates. Up to 50% of survivors show neurologic sequelae including hearing loss, cognitive impairments and learning disabilities, being particularly detrimental in affected infants and children where adjuvant therapy with dexamethasone has no proven beneficial effect. We evaluated the effect of concomitantly targeting specific pathophysiological mechanisms responsible for brain damage—i.e. matrix-metalloproteinase (MMP) activity and the exacerbated cerebral inflammation provoked through antibiotic-induced bacterial lysis. Here, we combined adjunctive therapies previously shown to be neuroprotective when used as single adjuvant therapies.

**Methods:**

Eleven-day-old Wistar rats were infected intracisternally with 6.44 ± 2.17 × 10^3^ CFU *Streptococcus pneumoniae* and randomised for treatment with ceftriaxone combined with (a) single adjuvant therapy with daptomycin (*n* = 24), (b) single adjuvant therapy with Trocade (*n* = 24), (c) combined adjuvant therapy (*n* = 66) consisting of daptomycin and Trocade, or (d) ceftriaxone monotherapy (*n* = 42). Clinical parameters and inflammatory CSF cytokine levels were determined during acute meningitis. Cortical damage and hippocampal apoptosis were assessed 42 h after infection. Morris water maze and auditory brainstem responses were used to assess neurofunctional outcome 3 weeks after infection.

**Results:**

We found significantly reduced apoptosis in the hippocampal subgranular zone in infant rats receiving adjuvant Trocade (*p* < 0.01) or combined adjuvant therapy (*p* < 0.001). Cortical necrosis was significantly reduced in rats treated with adjuvant daptomycin (*p* < 0.05) or combined adjuvant therapy (*p* < 0.05) compared to ceftriaxone monotherapy. Six hours after treatment initiation, CSF cytokine levels were significantly reduced for TNF-α (*p* < 0.01), IL-1β (*p* < 0.01), IL-6 (*p* < 0.001) and IL-10 (*p* < 0.01) in animals receiving combined adjuvant intervention compared to ceftriaxone monotherapy. Importantly, combined adjuvant therapy significantly improved learning and memory performance in infected animals and reduced hearing loss (77.14 dB vs 60.92 dB, *p* < 0.05) by preserving low frequency hearing capacity, compared to ceftriaxone monotherapy.

**Conclusion:**

Combined adjuvant therapy with the non-bacteriolytic antibiotic daptomycin and the MMP inhibitor Trocade integrates the neuroprotective effects of both single adjuvants in experimental paediatric pneumococcal meningitis by reducing neuroinflammation and brain damage, thereby improving neurofunctional outcome. This strategy represents a promising therapeutic option to improve the outcome of paediatric patients suffering from pneumococcal meningitis.

## Background

Pneumococcal meningitis (PM) causes considerable mortality and leads to long-lasting neurofunctional deficits in survivors [1, 2]. Neurofunctional sequelae from PM include hearing loss, epilepsy, cerebral palsy but also behavioural problems and cognitive deficits. Especially in affected children, these deficits negatively influence neurointegrative function, schooling performance and quality of life and may persist into adulthood [1–5]. Central nervous system (CNS) injury during PM is characterised by cortical necrosis and apoptosis of dentate gyrus granular cell progenitors in the hippocampus representing the histomorphologic correlates of behavioural and learning deficits [6–9] found in humans [10] and animal models [11, 12]. PM affects the peripheral nervous system (PNS) by damaging hair cells and spiral ganglion neurons in the inner ear leading to sensorineural hearing loss [13, 14]. Within the brain, neural cell death is caused by multiple factors including bacterial toxins and the excessive inflammatory host reaction [15–17]. Pneumolysin and hydrogen peroxide—produced by the pneumococci—are responsible for caspase-independent cell death in mature and immature dentate gyrus granular neurons [15–18]. Stimulated by pneumococcal components, leukocytes release soluble factors inducing a caspase-dependent apoptosis of immature neurons in the subgranular zone of the dentate gyrus [11, 15, 16]. Brain-resident immune cells, including microglia and leptomeningeal macrophages, are potently activated via TLR 2, 4 and 9 by pneumolysin, bacterial cell wall components and bacterial CpG DNA [15, 19, 20]. Together with the recruited neutrophils, activated microglia are able to produce large quantities of pro-inflammatory cytokines, reactive oxygen and nitrogen species, helping to eradicate the causative pathogen of PM but also contributing to the development of neuronal damage [15, 20, 21]. In addition, PM induces damage to hair cells and spiral ganglion neurons in the inner ear [13, 14, 22], provoking hearing impairments in up to 30% of survivors [2, 4, 23]. Increased levels of CSF TNF-α during acute PM has been positively correlated to increased hearing threshold in infant rat PM [13].

In clinical studies, adjunctive dexamethasone—an anti-inflammatory corticosteroid—was shown to improve the outcome of adults with PM in high-income countries and children with meningitis caused by *Haemophilus influenzae* type b [24]. To date, scientific evidence for the use of dexamethasone in children with PM is lacking. Moreover, in experimental models of PM, adjunctive dexamethasone aggravated mortality and acute hippocampal injury with subsequent learning performance deficits [9, 25–27]. Increased hippocampal apoptosis with adjuvant dexamethasone was also found independently in a rabbit model of *Escherichia coli* meningitis [28]. Adjuvant therapies targeting relevant pathophysiologic mechanisms in PM—such as non-bacteriolytic antibiotics, matrix metalloproteinase (MMP) inhibitors or antioxidants—have shown promising neuroprotective benefits in infant animal models [29–32]. Until now, most adjuvant therapies have been evaluated exclusively in the modality of single adjuvant therapies or combined with dexamethasone [33, 34], with very limited data on combined adjuvant interventions [35]. Based on present knowledge of the pathophysiology, we propose herein to combine complementary approaches targeting different pathophysiological mechanisms of PM, with the aim to more efficiently reduce CNS and PNS damage and improve the disease outcome. In this study, we evaluated a combination therapy consisting of daptomycin, a non-bacteriolytic antibiotic, with Trocade (Ro-32-3555, cipemastat), an orally active matrix metalloproteinase (MMP) inhibitor, originally designed to prevent cartilage breakdown in arthritis [36–39]. Single antibiotic therapy with daptomycin has previously been shown to clear pneumococci from CSF more rapidly than ceftriaxone without inducing bacterial lysis [40, 41]. In infant rat PM, adjunctive daptomycin with ceftriaxone reduced neuroinflammatory cytokine expression in the cerebrospinal fluid (CSF) and decreased brain injury compared to ceftriaxone monotherapy [29]. Trocade is an inhibitor of collagenases (MMP-1, -8 and -13) and gelatinase B (MMP-9) [36]. Adjuvant Trocade therapy in infant rat PM inhibited collagenase activity, decreased CSF levels of TNF-α and IL-1β, improved survival and reduced cerebral damage [30]. To our knowledge, this is the first study assessing behavioural outcomes after adjuvant daptomycin or Trocade therapy in experimental paediatric PM.

By targeting multiple pathophysiological mechanisms responsible for brain injury during acute bacterial meningitis with a combined adjunctive therapy regimen, we aim to integrate the neuroprotective effect of both substances and improve the neurofunctional outcome and quality of life after paediatric PM.

## Methods

### Infecting organism

A clinical isolate of *Streptococcus pneumoniae* (serotype 3) from a patient with bacterial meningitis was cultured overnight in brain heart infusion (BHI) medium, diluted 10-fold in fresh, pre-warmed BHI medium and grown for 5 h to reach the logarithmic phase as reported earlier [29, 42]. The bacteria were centrifuged for 10 min at 3100×*g*, washed twice and re-suspended in sterile, pyrogen-free saline (NaCl 0.85%). Bacteria were further diluted in saline to the desired density by measuring the optical density at 570 nm (OD_570_). Inoculum accuracy was determined by serial dilutions and plating on Columbia sheep blood agar (CSBA) plates.

### Infant rat model of pneumococcal meningitis

All animal studies were approved by the Animal Care and Experimentation Committee of the Canton of Bern, Switzerland (licence BE 129/14) and followed the Swiss national guidelines for the performance of animal experiments. A well-established infant rat model of PM was used for the experiments as previously described [29, 42]. Eleven-day-old Wistar rats together with their dams were obtained from Charles River Laboratories (Sulzfeld, Germany). The dams were provided with tap water and pellet diet at libitum. Litters were kept in rooms at a controlled temperature of 22 ± 2 °C. During the acute phase of the disease, animals were housed in conventional cages in a room with natural light. For long-term experiments after bacterial curing, animals were transferred to individually ventilated cages (IVC) in a room with controlled 12-h light/dark cycles. Intracisternal infections were performed by injection of 10 μl saline containing 6.44 ± 2.17 × 10^5^ CFU/ml living *S. pneumoniae*. Control animals received an equivalent volume of saline. Pneumococcal meningitis was confirmed 18 h post infection (hpi) by quantitative analysis of bacterial titres in CSF samples, when the animals developed symptomatic disease. For this, 5 μl of CSF were collected by puncture of the cisterna magna, followed by serial dilution and cultivation on a CSBA plates.

A total of 180 infant, mixed-sex rats were included in this study, representing 15 independent experiments with 12 infant rats per experiment (10 acute infection experiments plus 5 experiments to assess neurofunctional outcome). Specifically, 120 animals were included in the acute PM study to assess neuroinflammation and brain damage and an additional 60 animals were studied to investigate neurofunctional outcomes. Infected animals were randomised for treatment with ceftriaxone (CRO, 100 mg/kg, bid, i.p. Rocephine, Roche) combined with different adjuvant treatment groups: (a) single adjuvant therapy with daptomycin (DAP, *n* = 24, 10 mg kg^−1^ day^−1^, s.c. in saline, single application; Cubicin, Cubist Pharmaceuticals); (b) single adjuvant therapy with Trocade (*n* = 24, 2 × 75 mg kg^−1^ day^−1^, i.p in succinylated gelatine [Physiogel®, University Hospital Bern, Switzerland]); (c) combined adjuvant therapy using both DAP and Trocade (*n* = 66); (d) CRO monotherapy (*n* = 42, single s.c. saline application and i.p. succinylate gelatine, bid). Animals in the adjuvant daptomycin group received vehicle injections with Physiogel® (i.p.); animals in the adjuvant Trocade group received one saline vehicle injection (s.c.); and animals in the CRO monotherapy group received both vehicles. All animals received the same amount of fluids during the course of the experiment. All therapies were started at 18 hpi. Therapies involving DAP were started by the application of DAP followed by a 15-min delayed application (i.e. at 18:15 hpi) of all other therapies, similar to the currently recommended application sequence for adjuvant dexamethasone in adult PM [43]. Mock-infected animals either received combined adjuvant therapy (*n* = 12) or CRO monotherapy with vehicles (*n* = 12).

The rats were weighted and examined clinically at 0, 18, 24 hpi and before sacrificing at 42 hpi, as previously described [42]. Activity scores range from 1 = coma; 2 = does not turn upright; 3 = turns upright within 30 s; 4 = minimal ambulatory activity, turns upright in < 5 s; to 5 = normal. Investigators were blinded for treatment modalities. Spontaneous mortality was documented. Punctures of the cisterna magna were performed using a 30-gauge needle to obtain CSF samples at 18, 24, and 42 hpi. CSF samples not used for bacterial titre determination were centrifuged (16,000×*g* at 4 °C for 10 min), and supernatants were frozen at − 80 °C until further use.

Animals were sacrificed with an overdose of pentobarbital at 42 hpi and perfused with 4% paraformaldehyde (PFA) in phosphate-buffered saline (PBS) before their brains were removed and fixed in 4% PFA for histological analysis.

### Histomorphometric assessment of cortical damage and hippocampal apoptosis

In all animals sacrificed at 42 hpi, damage to cerebral structures was quantified as previously described by us and other independent research groups [11, 28, 30, 44–46]. Briefly, brains were fixed in 4% PFA and cryopreserved in 18% sucrose in PBS at 4 °C overnight. Coronal brain cryosections (45 μm thick) obtained by systematic uniform sampling were stained for Nissl substance with cresyl violet. Cortical damage was defined as areas of decreased neuronal density. Dead cells with histological features of apoptosis were quantified in 48 visual fields (× 400 magnification) spanning the hippocampus of both hemispheres. To prevent inter-rater confounding, histologic assessment was performed and evaluated by one single person blinded to treatment modalities of the individual animals.

### Quantitative analysis of cytokine expression in the CSF

A panel of cytokines previously found to be upregulated in PM [15, 47]—i.e. IL-1β, IL-6, TNF-α, IL-10 and IFN-γ—was assessed using a magnetic multiplex assay (Rat Magnetic Luminex® Assay, Rat Premixed Multi-Analyte Kit, R&D Systems, Bio-Techne) on a Bio-Plex 200 station (Bio-Rad Laboratories) as previously described [13, 30]. Five microlitres of CSF harvested and centrifuged at 18, 24 and 42 hpi was diluted to a final volume of 50 μl using the provided assay buffer. At least 50 beads were measured for each analyte. Calibration curves from recombinant standards were calculated with Bio-Plex Manager software (version 4.1.1) using a five-parameter logistic curve fitting. For samples below the detection limit, a value corresponding to the detection limit provided by the manufacturer (TNF-α, 22.1 pg/ml; IL-6, 56.0 pg/ml; IL-1β, 26.7 pg/ml; IL-10, 18.6 pg/ml; IFN-γ, 70.5 pg/ml) multiplied by the dilution factor was assigned.

### Assessment of learning and memory function by Morris water maze

Learning and memory performance was evaluated 3 weeks after infection using Morris water maze as previously described [48]. Swimming patterns of rats were recorded and evaluated with the video tracking system EthoVision XT-11 (Noldus Information Technology, Wageningen, Netherlands). The water was made turbid by addition of nontoxic black colour, and the water maze arena was virtually divided into four quadrants. An adjustable black platform measuring 16 × 13 cm was placed in the centre of the first quadrant 0.5 cm below the water surface, invisible to the swimming rat. Extra-maze distal cues were placed on the walls surrounding the water maze arena. Over 5 days, the animals performed five training trials per day with a hidden platform in a fixed position. Facing the tank wall, the rats were transferred into the water at one of the three entry zones determined by randomisation. When an animal found the platform within 90 s, it could stay on it for 15 s before starting the next training trial. If the rat did not find the platform within 90 s, it was guided to the platform by hand and could stay on it for 15 s. During training trials, latency to platform, distance to reach platform and velocity were recorded. On day 5 of the experiment, a probe trial without the platform was performed before and after the training trial session to assess long-term and short-term memory. Mean distance of rats to the centre of the virtual platform and percent of time spent in the quadrant that used to contain the platform were evaluated. Additionally, the first training trial per day was used as a measure of memory performance. On day 1, the first training trial was termed “day 0” for the learning curve (Fig. 4b). Investigators were blinded for treatment modalities of the individual animals. All measurements for swimming path were automatically recorded and computed by the video tracking system.

### Determination of hearing capacity by click and pure tone evoked auditory brainstem response

Auditory brainstem responses (ABRs) were recorded in response to click stimulations and pure tones on both ears using the SmartEP system (Intelligent Hearing Systems, Miami, USA), as previously described [13]. Animals were anesthetised with isoflurane (5% for induction and 2% for maintenance) using the Combi-Vet Vaporizer System equipped with a digital flowmeter (Rothacher Medical, Switzerland). One hundred-microsecond click stimuli and 5-ms pure tone pips (Blackman envelope; polarity alternating) were presented at a rate of 21.1 s^−1^, ranging from 100 to 20 dB SPL in 10 dB decrements (5 dB decrements close to threshold). Responses were measured at 4, 8, 16 and 32 kHz. A total of 1024 responses were averaged at each sound level and filtered between 100 and 1500 Hz. The hearing threshold was defined as the lowest intensity that induced the appearance of a visually detectable first peak. ABRs were recorded after water maze experiments (at postnatal day P37 to P39). Hearing thresholds were independently analysed by multiple blinded investigators.

### Statistical analysis

Statistical analyses were performed with GraphPad Prism (Prism 7; GraphPad Software Inc., San Diego, USA). If not stated otherwise, results are presented as mean values ± standard deviations. To compare data between two groups, an unpaired Student *t* test was used for parametric data and a Mann-Whitney for non-parametric data. Mortality rates were calculated using a log rank (Mantel-Cox) test for significance. For repeated measures (pure-tone hearing threshold, learning and memory performance in Morris water maze), a two-way ANOVA was performed to analyse differences between treatment modalities over time (for water maze data) or according to pure-tone frequencies (for ABRs). A two-tailed *p* value of < 0.05 was considered statistically significant, with *p* < 0.05 (*), *p* < 0.01 (**), *p* < 0.001 (***) and *p* < 0.0001 (****).

For exploratory data analysis, a multivariate linear regression model was used to estimate predictors and determinants for bacterial meningitis-induced hearing loss. The linear coefficients and a 95% confidence interval were calculated for each variable. These statistical analyses were performed using STATA 12 (STATA Corp., College Station, TX).

## Results

A total of 180 infant Wistar rats were included in the study. All animals infected with *S. pneumoniae* (*n* = 156) developed meningitis, as proven by growth of bacteria (≥ 10^7^ CFU/ml) in CSF samples obtained at 18 hpi and the appearance of disease symptoms (clinical score < 5, weight loss, changes in posture).

### Weight loss, clinical score and survival

**Fig. 1:**
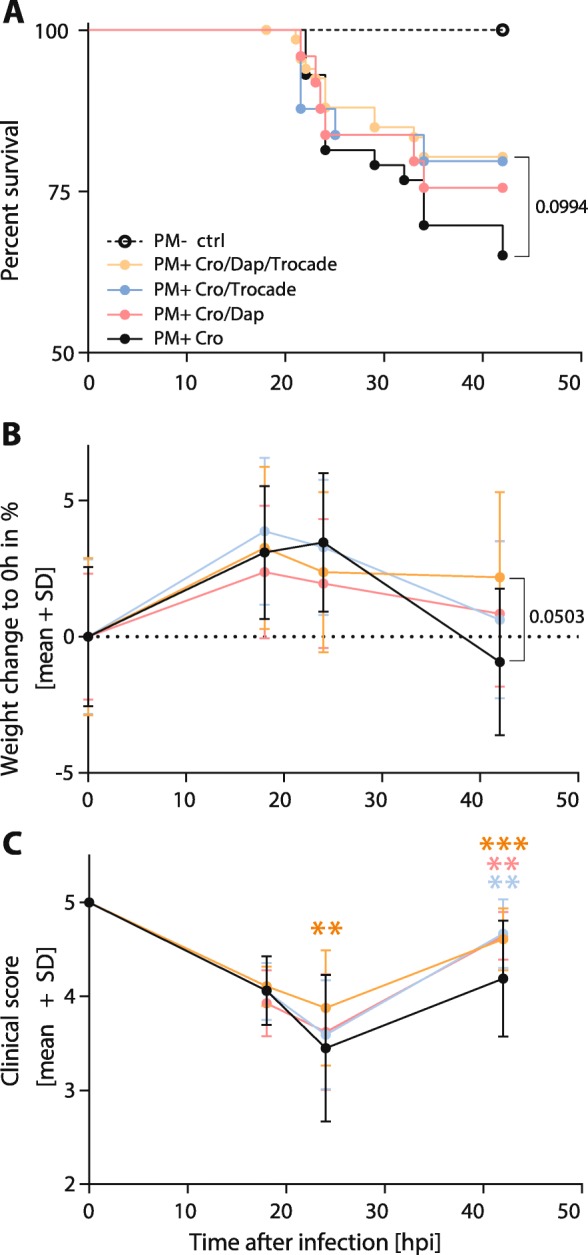
Survival, relative weight change and clinical scores of infant rats with acute pneumococcal meningitis. (**a**) Survival analysis revealed a significant difference of infected rats compared to mock-infected controls. Within the treatment groups, no significant differences in survival were found. Combined adjuvant therapy showed a non-significant trend (*p* = 0.0994) towards better survival compared to infant rats receiving CRO monotherapy. (**b**) Relative weight change during acute infections showed a slight increase within the first 18 hpi and a decrease of weight with the onset of clinical symptoms and treatment initiation until 42 hpi. In the combined adjuvant therapy group (Cro/Dap/Trocade), a strong trend points towards increased weight gain compared to animals receiving CRO monotherapy (Cro) at 42 hpi. (**c**) Clinical scores decrease upon infection until 24 hpi where the animals start to recover until 42 hpi. At 24 hpi, infant rats treated with combined adjuvant therapy displayed significantly higher clinical scores than animals treated with CRO monotherapy. Significance levels are always indicated as compared to CRO monotherapy

Survival was significantly reduced in infected animals (Fig. 1a) compared to mock-infected controls. Adjuvant therapies did not significantly improve survival compared to CRO monotherapy. Relative weight change in infected animals was not significantly altered by any of the treatment groups (Fig. 1b). However, the combined adjuvant therapy showed a strong trend towards improved weight gain at 42 hpi compared to CRO monotherapy (*p* = 0.0503). Upon infection, clinical scores in all infected animals decreased and reached a minimum at 24 hpi with subsequent improvement at 42 hpi. Clinical scores of infected infant rats treated with the combined adjuvant therapy were significantly improved compared to CRO monotherapy at 24 hpi (*p* < 0.01). At 42 hpi, all infected animals receiving adjuvant therapies demonstrated a significantly improved clinical score compared to CRO monotherapy, independent of the treatment regimen (*p* < 0.001 for CRO/DAP/Trocade; *p* < 0.01 for CRO/DAP; *p* < 0.01 for CRO/Trocade, Fig. 1c). Data on acute clinical parameters are summarised in Table 1.

**Table 1:** Relative weight change, clinical scores and bacterial CSF titres in infant rats with pneumococcal meningitis (*n* = 156) and uninfected controls (*n* = 24) during the acute phase of the disease. Activity scores represent 1 = coma; 2 = does not turn upright; 3 = turns upright within 30 s; 4 = minimal ambulatory activity, turns upright in < 5 s; and 5 = normal. Weight change describes percentage of weight gain compared to weight at time of infection. Bacterial titre depicts colony-forming units (CFU) per millilitre CSF at 18 hpi. Data represent mean ± SD. hpi hours post infection, PM pneumococcal meningitis, Cro ceftriaxone, Dap daptomycin

	18 hpi			24 hpi		42 hpi	
	Activity	Weight change	Bacterial titre	Activity	Weight change	Activity	Weight change
PM+ Cro (*n* = 42)	4.1 ± 0.4	3.1 ± 2.4	1.46 ± 1.29 × 10^8^	3.4 ± 0.8	3.5 ± 2.5	4.2 ± 0.6	− 0.9 ± 2.7
PM+ Cro+Dap (*n* = 24)	4.1 ± 0.3	3.8 ± 2.7	1.03 ± 0.72 × 10^8^	3.6 ± 0.6	3.3 ± 2.9	4.6 ± 0.3	0.6 ± 2.9
PM+ Cro+Trocade (*n* = 24)	3.9 ± 0.3	2.4 ± 2.4	0.96 ± 0.56 × 10^8^	3.6 ± 0.6	2.0 ± 2.4	4.7 ± 0.4	0.8 ± 2.7
PM+ Cro+Dap+Trocade (*n* = 66)	4.1 ± 0.3	3.3 ± 3.0	1.15 ± 0.76 × 10^8^	3.9 ± 0.6	2.4 ± 3.0	4.7 ± 0.3	2.2 ± 3.1
PM− ctrl (*n* = 24)	5.0 ± 0	13.8 ± 2.6	Undetectable	5.0 ± 0	21.8 ± 2.8	4.98 ± 0.1	31.1 ± 5.8

### Combined adjuvant therapy reduces hippocampal apoptosis and cortical necrosis

**Fig. 2:**
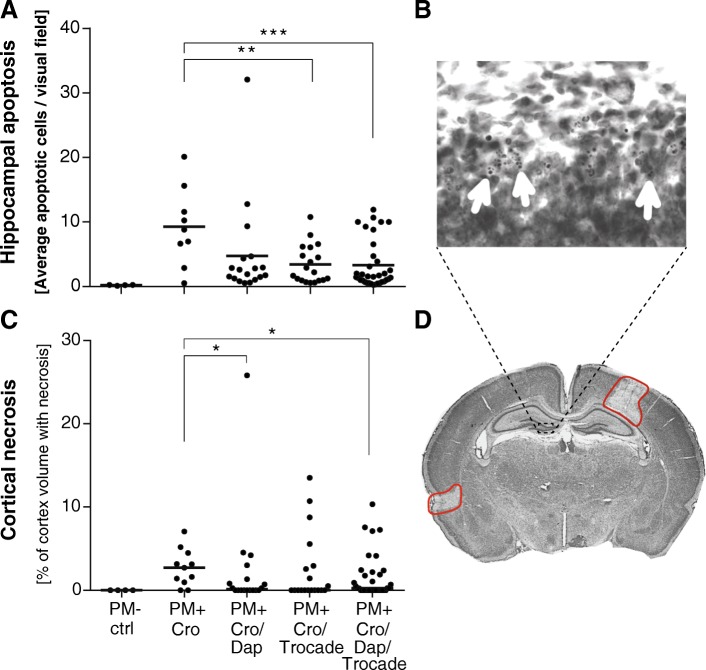
Hippocampal apoptosis and cortical damage during acute pneumococcal meningitis. Hippocampal apoptosis was most abundant in infant rats receiving CRO monotherapy. (**a**) Treatment with adjuvant Trocade and with combined adjuvant therapy significantly reduced hippocampal apoptosis. (**b**) Apoptotic cells in the subgranular zone of the dentate gyrus show condensed nuclei or formation of apoptotic bodies (white arrows). (**c**) Cortical necrosis was most prominently observed in animals receiving CRO monotherapy. Adjuvant daptomycin and combined adjuvant therapy significantly reduced cortical necrosis. (**d**) Cortical necrosis with reduced neuronal density is visible on cresyl violet stained brain sections. Foci of cortical necrosis are indicated by red lines. In **a**, the horizontal line depicts the sample mean and an unpaired *t* test was used for single comparison. In **c**, the horizontal line represents the sample median and a Mann-Whitney test was used for single comparison

In mock-infected animals (PM-), hippocampal apoptosis was found to occur at physiologic levels and no cortical damage was detectable (Fig. 2a, c), as previously described [9]. The highest amount of apoptotic neurons in the subgranular zone of the dentate gyrus was found in animals receiving CRO monotherapy (9.26 ± 6.06 apoptotic cells per visual field, *n* = 9). Hippocampal apoptosis was significantly reduced in infant rats receiving adjuvant Trocade (3.41 ± 2.97 apoptotic cells per visual field, *n* = 19, *p < 0.01*) and in animals receiving the combined adjuvant therapy (3.307 ± 3.70 apoptotic cells per visual field, *n* = 35, *p* < 0.001, Fig. 2a) compared to animals treated with CRO monotherapy. Adjuvant therapy with DAP did not significantly reduce hippocampal apoptosis (4.75 ± 7.52 apoptotic cells per visual field, *n* = 18, *p* = 0.131).

The highest extent of cortical necrosis was found in the animals receiving CRO monotherapy (2.70% [IQR 0.97–4.49]). Cortical necrosis was significantly reduced in infant rats treated with adjuvant DAP (0.13% [IQR 0–1.75], *p* < 0.05) and in animals receiving the combined adjuvant therapy (0.25% [IQR 0–2.19], *p* < 0.05, Fig. 2c). In this study, adjuvant Trocade did not significantly decrease the amount of cortical necrosis in infant rats with pneumococcal meningitis. The range of hippocampal apoptosis and cortical necrosis were found at levels of previously published studies [29, 30, 48].

As only the combined adjuvant therapy was able to protect from both acute cerebral damages, further analysis therefore focused on comparing the combined treatment modality to CRO monotherapy.

### Combined adjuvant therapy decreases inflammation and clears bacteria faster in CSF

**Fig. 3:**
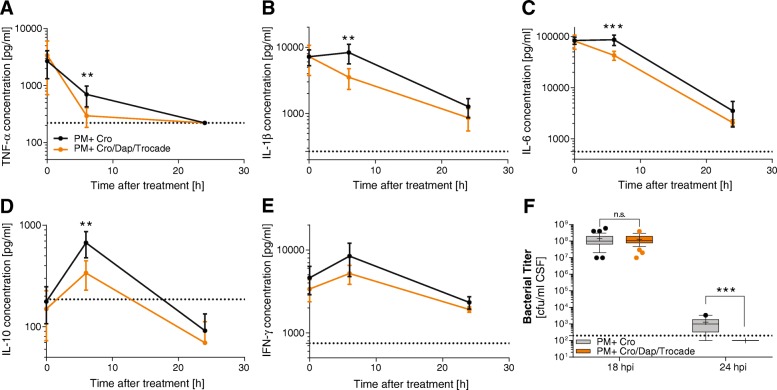
Inflammatory cytokines and bacterial clearance in the CSF during the acute pneumococcal meningitis. Cytokine levels are represented by mean ± 95% confidence interval starting before treatment initiation, 6 h and 24 h after treatment start (representing 18, 24 and 42 hpi) for TNF-α (**a**), IL-1β (**b**), IL-6 (**c**), IL-10 (**d**) and IFN-γ (**e**). Six hours after treatment initiation, TNF-α, IL-1β, IL-6 and IL-10 were significantly reduced in infected animals receiving the combined adjuvant therapy compared to infected animals with CRO monotherapy. Bacterial titres in the CSF were similar in both treatment groups before starting therapy (at 18 hpi) with a faster bacterial clearance in animals receiving the combined adjuvant therapy (**f**). Statistical differences were assessed using an unpaired t-test for cytokines and bacterial titre at 18 hpi. For bacterial titre at 24 hpi, a Mann-Whitney test was used as data were not normally distributed

Inflammatory cytokines were measured immediately before, 6 and 24 h after treatment initiation (representing 18, 24 and 42 hpi). At treatment start, there was no significant difference in the cytokine profile between infected animals receiving CRO monotherapy or combined adjuvant therapy, indicating a comparable severity of inflammation between these two groups (Fig. 3a–e). Six hours after treatment initiation, levels of TNF-α (*p* < 0.01), IL-1β (*p* < 0.01), IL-6 (*p* < 0.001) and IL-10 (*p* < 0.01) were significantly reduced in animals receiving combined adjuvant therapy (*n* = 14) compared to CRO monotherapy (*n* = 15). Thereafter, all CSF cytokine levels decreased and no differences in CSF cytokine concentrations were found between the two groups 24 h after starting the therapy. Similar bacterial CSF concentrations were found in both groups before treatment initiation (1.46 ± 1.29 × 10^8^ CFU/ml for CRO (*n* = 38) vs. 1.31 ± 0.86 × 10^8^ CFU/ml for CRO/DAP/Trocade (*n* = 38), *p* = 0.566, Fig. 3f), confirming a comparable severity of infection. Six hours after therapy start, bacterial CSF concentrations were efficiently reduced in both treatment groups. While none of the animals with combined adjuvant therapy showed detectable bacteria in the CSF (< 10^2^ CFU/ml, *n* = 11), the majority of animals treated receiving CRO monotherapy still presented living bacteria in the CSF (median of 1.00 × 10^3^ CFU/ml vs. 1.00 × 10^2^ CFU/ml, *n* = 10, *p* < 0.001), indicating faster bacterial clearance in the CSF in animals receiving combined adjuvant therapy compared to monotherapy with CRO.

### Combined adjuvant therapy improves learning and memory performance

**Fig. 4:**
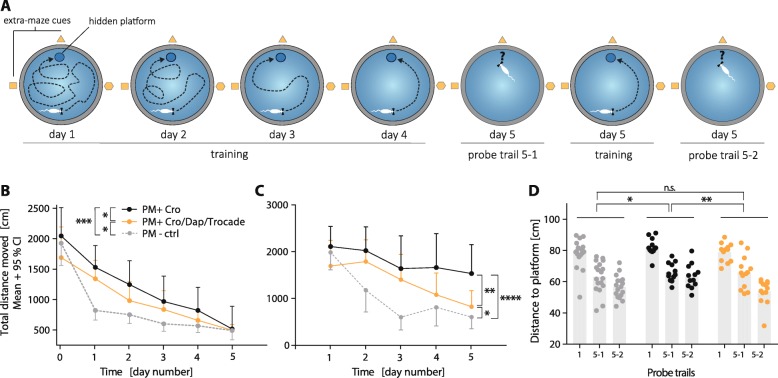
Learning and memory performance 3 weeks after acute pneumococcal meningitis assessed by Morris water maze. (**a**) Schematic representation of our Morris water maze protocol used during these experiments (scheme sketch inspired by Kipnis et al. [35]). Learning performance assessed on five consecutive days revealed impaired learning of infected animals compared to mock-infected animals. Infected animals treated with combined adjuvant therapy revealed significantly improved learning behaviour compared to their infected counterparts receiving CRO monotherapy (**b**). Memory assessment—by analysing the first training trial for each day separately—showed significantly reduced memory performance in infected animals compared to mock-infected animals. Infected animals treated with combined adjuvant therapy showed significantly improved memory compared to their infected counterparts treated with CRO monotherapy (**c**). Memory formation assessed with probe trials after removing the platform further demonstrated the impaired performance of infected animals with CRO monotherapy to infected animals receiving combined adjuvant therapy and to mock-infected animals (**d**). Statistical differences were assessed by two-way ANOVAs to compare learning and memory performance between groups over repeated time points

Neurofunctional outcome after pneumococcal meningitis was evaluated using a Morris water maze test (Fig. 4a) 3 weeks after infection. Learning ability was observed in all animal groups, as indicated by a steady decrease of total distance swum until reaching the platform during the training trials over the 5 days (Fig. 4b). Mock-infected animals (PM− ctrl, *n* = 20) showed significant better learning performance than infected animals receiving CRO monotherapy (PM+ CRO, *n* = 12, *p* < 0.001) and infected animals receiving combined adjuvant therapy (PM+ CRO/DAP/Trocade, *n* = 13, *p* < 0.05). Animals receiving combined adjuvant therapy performed significantly better in the learning assessment than infected animals with CRO monotherapy (*p* = 0 < 0.05). Analysing latency to reach platform during training trials revealed similar findings for learning performance (data not shown).

Mock-infected animals showed the best performance for memory formation over the 5 days of testing, as assessed by separate analysis of the first training trial per day (Fig. 4c). These animals displayed significant better memory compared to infected animals receiving CRO (*p* < 0.0001) and to infected animals receiving combined adjuvant therapy (*p* < 0.05). Infected animals receiving combined adjuvant therapy showed significantly improved memory performance compared infected animals receiving CRO monotherapy (*p* < 0.01). On days 1 and 5, spatial memory was further assessed using probe trials performed without platform by evaluating the average distance of the animal’s swimming path to the virtual platform location. Two-way ANOVA tests revealed impaired performance of infected animals receiving CRO monotherapy to mock-infected animals (*p* < 0.05) and to infected animals receiving combined adjuvant therapy (*p* < 0.01, Fig. 4d). For the second probe trial performed on day 5—representing short-term memory formation—a significant better performance of infected animals receiving the combined adjuvant therapy compared to infected animals receiving only CRO (*p* < 0.01) was documented. Analysing probe trial memory formation by time spent in the quadrant that used to harbour the platform revealed similar findings (data not shown).

### Combined adjuvant therapy reduces hearing loss

**Fig. 5:**
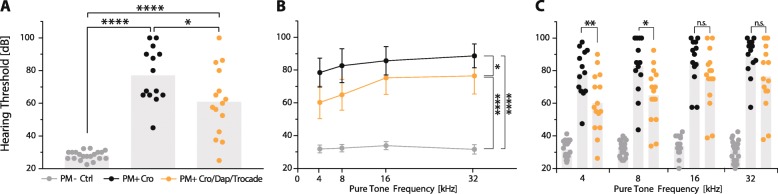
Hearing capacity assessed 3 weeks after acute pneumococcal meningitis. ABR recordings using broadband click stimuli disclosed significantly increased hearing threshold in infected rats. Combined adjuvant therapy significantly improved hearing capacity in infected rats compared to CRO monotherapy (**a**). Pure tone ABR threshold (mean ± 95% confidence interval) demonstrated significantly impaired hearing in infected animals but improved hearing function in infected rats receiving the combined adjuvant therapy (**b**). Frequency-specific hearing threshold in infected rats revealed preservation of low frequency hearing (4 kHz and 8 kHz) in rats receiving the combined adjuvant therapy (**c**). Statistical differences were assessed by single comparison using an unpaired *t* test in **a** and **c** and by two-way ANOVA in **b**

Hearing thresholds were determined 4 weeks after infection using broadband click stimuli and pure tones. We found significantly increased hearing thresholds in infected animals compared to their respective mock-infected control animals (CRO-treated animals 77.14 ± 16.84 dB vs. 28.38 ± 1.67 dB in mock-infected controls, *p* < 0.0001; combined adjuvant therapy-treated animals 60.92 dB ± 20.82 vs. 27.85 ± 3.55 dB in mock-infected controls, *p* < 0.0001, Fig. 5a). In infected rats, combined adjuvant therapy significantly improved average hearing capacity compared to CRO monotherapy (*p* < 0.05). Pure tone ABR testing revealed higher thresholds for all assessed frequencies in infected animals compared to mock-infected animals (*p* < 0.0001, two-way ANOVA, independent of therapeutic modality, Fig. 5b). Infected animals that received combined adjuvant therapy demonstrated significantly better pure tone hearing capacity than those treated with CRO monotherapy (*p* < 0.05, Fig. 5b). Frequency-specific analysis disclosed that at lower frequencies (4 kHz and 8 kHz), hearing was improved in animals treated with combined adjuvant therapy (Fig. 5c) 60.25 ± 17.9 dB vs 78.46 ± 14.49 dB for 4 kHz (*p* < 0.01) and 64.92 ± 16.99 dB vs 82.69 ± 17.24 dB for 8 kHz (*p* < 0.05). No significant difference was found at 16 kHz and 32 kHz.Univariate linear regression analysis (Table 2) revealed significantly increased hearing thresholds with increasing bacterial titres in CSF (29.5 dB per additional log of CSF titres at 18 hpi, *p* < 0.01). Treatment with combined adjuvant therapy (− 16.8 dB compared to infected animals treated with CRO monotherapy, *p* < 0.05) and better clinical score at 24 hpi (− 21.7 dB per additional score, *p* < 0.01) were predictive for reduced hearing loss. The multivariate linear regression analysis—including bacterial titres in the CSF at 18 hpi and treatment regimen—presented a significant contribution of bacterial titres to higher hearing threshold (35.1 dB per additional log of CSF titre at 18 hpi, *p* < 0.001) and a significant benefit of treating infected animals with combined adjuvant therapy (− 21.7 dB compared to infected animals receiving CRO monotherapy, *p* < 0.001).

**Table 2:** Univariate and multivariate linear regression for click hearing threshold in infant rats (*n* = 29) 4 weeks after surviving an episode of pneumococcal meningitis measured by click stimuli

	Hearing threshold					
	Univariate			Multivariate		
	Coef.	95% CI	*p*	Coef.	95% CI	*p*
Log bacterial CSF titre at 18 hpi	29.5	11.13–47.8	0.003	35.1	20.1–50.0	< 0.001
Treatment with combined adjuvant therapy	− 16.8	− 31.3 to − 2.2	0.026	− 21.7	− 32.7 to − 10.7	< 0.001
Female sex	1.8	− 15.5–19.1	0.832	–	–	–
Relative weight gain (per add. percent)	− 1.03	− 2.4–0.4	0.141	–	–	–
Clinical score at 24 hpi	− 21.7	− 35.4 to − 7.9	0.003	–	–	–

## Discussion

The results of the present study demonstrate that a combined adjuvant therapy consisting of DAP and Trocade was able to integrate the beneficial effects of both compounds in experimental paediatric PM. Moreover, the adjuvant combination therapy significantly improved the neurofunctional outcome in experimental infant rat PM compared to CRO monotherapy.

Brain damage caused by PM is associated with an exacerbated CSF inflammation and results in an unfavourable neurofunctional outcome of the disease [15, 49–51]. The initial unrestricted growth of *S. pneumoniae* in CSF produces large amounts of pathogen-associated molecular patterns (PAMPs), a situation that is aggravated by treatment with bacteriolytic antibiotics, causing release of bacterial components into the CSF and inducing an even more severe inflammatory reaction [49, 50]. Based on these observations, the use of adjunctive therapies is meant to attenuate this cerebral hyperinflammatory reaction. In adults and children with PM, dexamethasone is currently recommended as adjuvant therapy in high-income countries based on a Cochrane meta-analysis [24, 43]. The beneficial effect of corticosteroids in paediatric bacterial meningitis was, however, exclusively demonstrated for meningitis caused by *Haemophilus influenzae* and not for PM or other causative pathogens [24]. In animal models for infant PM, the outcome after adjunctive dexamethasone therapy was aggravated with regard to survival, weight loss, hippocampal apoptosis and memory performance [9, 25, 26]. Since this present study was performed in a model of infant PM, we did not compare treatment regimens to adjunctive dexamethasone, which has previously been shown to impair the outcome in comparison to CRO monotherapy [9, 25–28].

Daptomycin, a non-bacteriolytic and bactericidal lipopeptide antibiotic, is used for treatment of serious Gram-positive infections [52]. Insertion of the lipophilic tail of daptomycin into the bacterial cell membrane causes a rapid membrane depolarisation, thereby stopping biosynthesis and causing bacterial death [52–54]. Treatment with daptomycin has been shown to minimise antibiotic-induced cell wall lysis compared to ceftriaxone in experimental pneumococcal meningitis in rabbits [40]. In previous work, we showed that daptomycin in infant rat PM led to a faster bacterial clearance from the CSF, thereby reducing inflammatory parameters and decreasing cortical injury compared to CRO treatment [41, 55]. Addition of daptomycin to CRO in infant rats reduced PM-associated cerebral damage, inflammatory cytokine levels in the CSF and improved hearing capacity [29]. In adult rats with PM, daptomycin treatment attenuated cognitive impairment compared to CRO [56]. Although *S. pneumoniae* and group B streptococci (GBS) are very frequent causes of paediatric and neonatal bacterial meningitis, recent European data reported that *Neisseria meningitidis* and *Haemophilus influenzae* account for 55% of all paediatric cases beyond neonatal age [43]. Thus, for empiric therapy, daptomycin needs to be combined with a broad spectrum antibiotic targeting Gram-negative bacteria (e.g. CRO as an expanded-spectrum cephalosporin), to cover potential infection with *N. meningitidis* or *H. influenzae* [29].

Brain injury in acute PM involves an upregulation of MMP activity, measurable in the CSF of infected patients [15, 57] with higher MMP-9 level being associated with the development of hearing impairment or secondary epilepsy in infected children [57]. MMP-9 weakens the blood-brain barrier (BBB) by degrading collagen, e.g. in the basal lamina [42, 58], thereby facilitating leukocyte extravasation and BBB leakage [30, 59]. Moreover, MMPs are potent sheddases and convertases able to cleave and activate inflammatory cytokines and chemokines, thereby contributing to the hyperinflammatory reaction driving the development of brain damage [30, 60–62]. In previous work, we showed that MMP inhibition with adjunctive Trocade treatment in infant rats with PM increased survival and significantly reduced the collagenase activity of MMPs, the intrathecal concentrations of pro-inflammatory cytokines and the level of cortical necrosis and hippocampal apoptosis [30].

### Combining adjuvant daptomycin and Trocade reduces CSF inflammation and neuropathology in experimental paediatric PM

During acute PM, combining adjuvant daptomycin and Trocade improved clinical scores of infant rats at 24 and 42 hpi and showed a non-significant trend (*p* = 0.0503) towards improved weight gain at 42 hpi compared to CRO monotherapy (Fig. 1b). Single adjuvant therapies with either daptomycin or Trocade also improved the clinical scoring at 42 hpi. In the present study, no significant difference in survival could be found between groups. In an earlier report with adjuvant Trocade therapy, survival was improved [30]. In this earlier study, however, adjuvant Trocade therapy was initiated at an earlier time point after infection (at 5 hpi), whereas in the present study, it was applied concomitantly with the appearance of the clinical symptoms (at 18 hpi) and the initiation of antibiotic treatment.

All tested adjuvant therapies protected the subjects from at least one form of neuronal damage observed during pneumococcal meningitis (Fig. 2). Adjuvant Trocade significantly reduced the amount of apoptosis in the dentate gyrus of the hippocampus. On the other hand, adjuvant daptomycin significantly reduced cortical necrosis. The adjuvant combination therapy was superior to single adjuvants by reducing both forms of cerebral damage. Thereby, we could validate our hypothesis that adjuvant therapies targeting different pathophysiological mechanisms during acute PM generate additive protective effects on brain damage. The neuroprotective effects of single adjunctive Trocade and daptomycin have already been reported previously [29, 30]. Since only the combined adjuvant therapy was able to significantly reduce both forms of brain injury during acute PM, we further focused on comparing the combination therapy to the CRO monotherapy. Combining adjuvant daptomycin and Trocade with CRO reduced CSF levels of TNF-α, IL-1β, IL-6 and IL-10 6 hours after treatment initiation and caused a faster bacterial clearance in the CSF (Fig. 3). Faster bacterial clearance might be attributed to adjuvant daptomycin, as reported earlier [40, 41, 55]. Overall reduction in neuroinflammatory parameters is mostly likely caused by a combined effect of faster bacterial clearance and limited release of bacterial cell wall components due to daptomycin [40, 41, 55] plus reduced collagenase-induced BBB leakage and reduced cytokine and chemokine shedding by Trocade [30, 60–62]. In the present study, we found no statistically significant reduction of IFN-γ CSF levels in infected animals treated with combined adjuvant therapy compared to CRO monotherapy. This is in agreement with findings from previous studies on single adjuvant Trocade therapy and single antibiotic therapy with daptomycin that did not show significantly reduced IFN- γ levels [30, 55]. Differences in inflammatory cytokine reduction, bacterial clearance and clinical parameters are in accordance to what has been previously reported for single adjuvant therapies [13, 29, 30, 40, 41, 55].

### Combining adjuvant daptomycin and Trocade improves the neurofunctional outcome in experimental paediatric PM

Hippocampal apoptosis during acute pneumococcal meningitis correlates with impaired learning performance and memory formation [6–9], in turn preservation of the dentate gyrus is associated with intact learning and memory performance [9, 48]. Combining adjuvant daptomycin and Trocade preserved learning and memory performance assessed by Morris water maze testing (Fig. 4b). Compared to uninfected control animals, infected rats, independently of the treatment regimen, showed significantly decreased learning performance. However, infected rats receiving combined adjuvant therapy showed significantly improved learning performance compared to their infected counterparts receiving CRO monotherapy. Memory performance testing revealed that infected animals treated with CRO monotherapy presented markedly impaired spatial memory compared to uninfected animals (Fig. 4c). Animals receiving combined adjuvant therapy showed significantly improved memory performance compared to infected rats receiving CRO monotherapy, although demonstrating significant memory impairments compared to uninfected controls. Probe trials performed on day 5 confirmed that infected animals treated with CRO monotherapy had impaired memory formation compared to uninfected animals. These memory impairments were prevented by adjuvant combination therapy (Fig. 4d). Short term memory—assessed with the second probe trial—was especially improved in animals receiving combined adjuvant therapies. By reducing hippocampal apoptosis, combined adjuvant therapy thus improved learning and memory formation. As long-term memory consolidation and retrieval is a complex procedure and also involves cortical structures [63–65], reduction of cortical necrosis during acute PM possibly also contributed to the improved learning and memory performance found in animals receiving combined adjuvant therapy.

During acute PM, pneumococci and leukocytes infiltrate the cochlea from the CSF via the cochlear aqueduct [2, 4, 13, 23, 66], causing damage to the spiral ganglion neurons and sensory hair cells, resulting in sensorineural hearing loss [13, 14, 22, 25, 67–69]. Previous studies showed that TNF-α levels during acute experimental PM positively correlate with the severity of hearing loss [13] and that a reduction in inflammatory cytokines improved hearing capacity [29, 55, 67]. Here, combining daptomycin with Trocade during acute PM significantly reduced inflammatory cytokines and improved hearing capacity 4 weeks after infection (Fig. 5). Frequency-dependent analysis showed that hearing at low frequencies was preserved, which would correspond to a protection of cochlear sensory hair cells and spiral ganglion neurons in the cochlear apex. As bacteria from the CSF enter the inner ear via the cochlear aqueduct, we would expect a gradient of ototoxic substances with highest concentrations at the base and decreasing towards the apex—a hypothesis supported by previous animal and human studies [13, 70]. Our results suggest that faster bacterial clearance together with overall reduction of inflammatory parameters may reduce the concentration of ototoxic substances particularly in the apex thereby preserving hearing capacity at low frequencies. This finding is in line with an earlier study showing that the severity of infection modulates the extent of hearing loss, with moderately infected animals having a preserved hearing capacity for low frequencies perceived at the cochlear apex, whereas severely infected animals lost hearing capacity at all frequencies [13]. Multivariate linear regression confirmed a beneficial effect of the combination therapy on hearing loss after adjusting for severity of infection based on bacterial CSF titre at 18 hpi. Combined adjuvant therapy was able to significantly preserve 21.7 dB hearing capacity compared to CRO monotherapy (Table 2).

Limitations of this study include lack of comparison to single adjuvants in long-term experiments. As only combined adjuvant therapy was able to reduce both forms of cerebral damage, i.e. cortical necrosis and hippocampal apoptosis, behavioural tests were only conducted in infected animals receiving combined adjuvant therapy or CRO monotherapy. Thus, we do not have a direct comparison to single adjuvant therapies for neurofunctional outcomes. The proven superiority of combined adjuvant therapy to single adjuvant therapies is therefore based on data gathered from the acute phase of the disease. Furthermore, we did not include a direct comparison to adjuvant dexamethasone, as there is no clear evidence for its beneficial effect on the outcome of paediatric PM in the clinics [24, 71] nor animal models [9, 25–28].

## Conclusion

Combined adjuvant therapy with non-bacteriolytic daptomycin and the matrix-metalloproteinase inhibitor Trocade reduces CSF cytokine levels, known to be mediators of brain damage during acute PM. Neuroprotective effects of single adjuvants were integrated by combined adjuvant intervention and attenuated neurologic sequelae by preserving learning and memory performance and preventing hearing loss. Based on these results, we conclude that combining adjuvant daptomycin and Trocade with ceftriaxone is a promising therapeutic option to improve the outcome of paediatric PM.

## Abbreviations

ABR: Auditory brainstem response
BBB: Blood-brain barrier
BHI: Brain heart infusion
CFU: Colony-forming units
CNS: Central nervous system
CRO: Ceftriaxone
CSF: Cerebrospinal fluid
DAP: Daptomycin
GBS: Group B streptococci
MMP: Matrix-metalloproteinase
MWM: Morris water maze
PM: Pneumococcal meningitis
PNS: Peripheral nervous system
